# Supplementary material for: Co-infection of the four major Plasmodium species: Effects on densities and gametocyte carriage
Source: PLoS Negl Trop Dis. 2022 Sep 13;16(9):e0010760. doi: 10.1371/journal.pntd.0010760 (PMC9506632; doi:10.1371/journal.pntd.0010760)
Supplement: S1 Text — (DOCX) [file pntd.0010760.s001.docx]

**Co-infection of the four major *Plasmodium* species: effects on densities and gametocyte carriage**

**Appendix and Supplementary Methods**

Aurel Holzschuh, Maria Gruenberg, Natalie E. Hofmann, Rahel Wampfler, Benson Kiniboro, Leanne J. Robinson, Ivo Mueller, Ingrid Felger, Michael T. White

***Correspondence to Dr Michael White:** [**michael.white@pasteur.fr**](mailto:michael.white@pasteur.fr)

1. **CONSORT diagram**

**
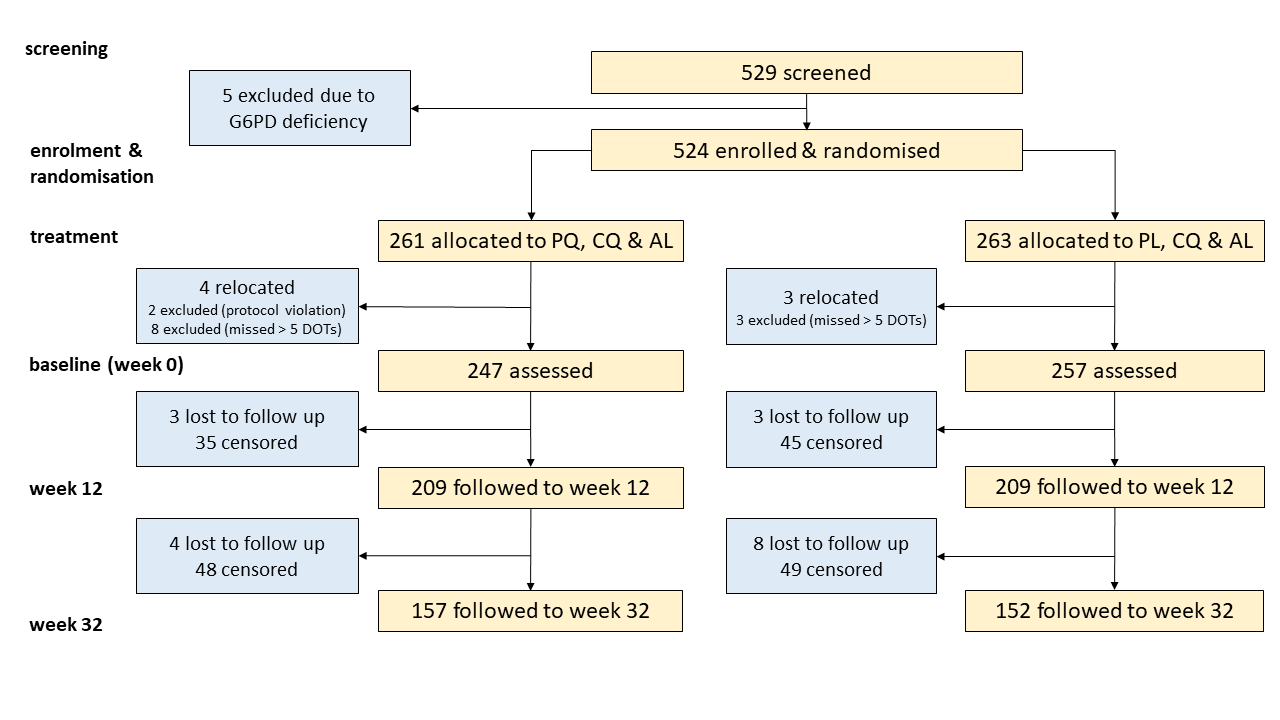
**

**Figure A: Consort diagram.** Study design, randomisation, and retention of study participants during follow-up. In the analysis of time to first infection and clinical episode, children were censored on the last day before the first of two consecutive missed clinical visits. Based on data initially published in Robinson et al (1)**.**

1. **Review of *Plasmodium* co-infection literature**

**Table A. Interaction between asexual parasite stages of different Plasmodium species.**

| ***Plasmodium* species** | **Area of study** | **Year of study** | **Diagnostic method** | **Findings** | **Reference** |
| --- | --- | --- | --- | --- | --- |
| *Pf, Pv, Pm, Po** | Africa, Asia, Europe, Oceania, North America, South America | 1923-1999 | microscopy and other | (i) 22 significant *Pf-Pm* associations, 14 of which were positive (in Africa) or negative (in Asia). (ii) 26 significant *Pf-Pv* associations, 17 of these were negative in Asia, but positive in Tanzania and PNG. (iii) Significant association for *Po* was always positive with either *Pf* or *Pm* | Howard *et al.*, 2001 (2) |
| *Pf-Pm;*  *Pf-Po* | Ghana | 1998 | microscopy | Higher odds of *Pm* (OR=2.63) or *Po* (OR=2.02) infections if co-infected with *Pf.* | Browne *et al.*, 2000 (3) |
| *Pf-Pm* | Nigeria | 1970-1972 | microscopy | (i) *Pf* or *Pm* infections are more likely in the presence than in the absence of the other species. (ii) Individuals with higher *Pf* densities are more likely to be co-infected with *Pm*. | Molineaux *et al.*, 1980 (4) |
| *Pf-Pm-Po;*  *Pf-Pm-Pv*† | Africa, South Asia, Southeast Asia, Southwest Pacific | 1984-1997 | microscopy | (i) Significant surplus of *Pf-Pm-Po* co-infections. (ii) Significant deficit of *Pf-Pm-Pv* co-infections. | McKenzie *et al.*, 1999 (5) |
| *Pf-Pm;*  *Pf-Po* | Mozambique | 2001-2002 | PCR | (i) Almost all *Pm* and *Po* infections occur as co-infections with Pf. (ii) Decreased *Pm* prevalence results in increased *Pf* prevalence. | Marques *et al.* 2005 (6) |
| *Pf-Pm;*  *Pf-Po* | DRC | 2013-2014 | qPCR | Almost all *Pm* and *Po* infections occur as co-infections with *Pf.* | Doctor *et al.*, 2016 (7) |
| *Pf-Pm;*  *Pf-Po* | Gabon | 2016 | qPCR | Almost all *Pm* and *Po* infections occur as co-infections with *Pf.* | Woldearegai *et al.*, 2019 (8) |
| *Pf-Pm* | Ivory Coast | N/A | PCR | *Pf-Pm* co-infections are more prevalent in asymptomatic individuals (27%) than in symptomatic individuals (0%). *Pm* is protective against *Pf* disease. | Black *et al.*, 1994 (9) |
| *Pf-Pm;*  *Pf-Pv* | PNG | 1990-1992 | microscopy | Protective effect of *Pv* and *Pm* against *Pf* disease in *Pf-Pv* and *Pf-Pm* co-infections. | Smith *et al.* 2001 (10) |
| *Pf-Pv*^‡^ | Asia, Africa, South America | 1975-2001 | microscopy | Infection with *Pf* or *Pv* decreases the risk of infection with the other species (i.e. suppression). | Haghdoost *et al.*, 2007 (11) |
| *Pf-Pv* | Vanuatu | 1993-1996 | microscopy | α+-thalassemic young children with *Pf-Pv* co-infections show a reduced risk of severe malaria compared to *Pf* mono-infections. | Williams *et al.*, 1996 (12) |
| *Pf-Pm* | Ghana | N/A | PCR | α+-thalassemic individuals with *Pf-Pm* co-infections show a lower risk of severe malaria than *Pf* mono-infections. | Mockenhaupt *et al.*, 2001 (13) |
| *Pf-Pm* | Kenya | 2008-2016 | qPCR | Reduced risk of fever in *Pf-Pm* co-infections compared to *Pf* mono-infections (aOR=0.43). | Akala *et al.* 2021 (14) |
| *Pf-Pv* | Thailand | 1990-1992 | microscopy | Reduced risk of severe malaria in *Pf-Pv* co-infections (5.7%) compared to *Pf* mono-infections (1.6%). | Luxemburger *et al.*, 1997 (15) |
| *Pf-Pv* | Thailand | 1990-1995 | microscopy | Reduced risk of severe anemia in *Pf-Pv* co-infections compared to *Pf* mono-infections. | Price *et al.*, 2001 (16) |
| *Pf-Pv* | Thailand | 1993-1995 | microscopy | Decreased risk of treatment failure in patients with *Pf-Pv* co-infections compared to *Pf* mono-infections. | Price *et al.*, 1997 (17) |
| *Pf-Pv* | Thailand | 1998, 1999, 2001 | microscopy | More severe illness in *Pf-Pv* co-infections compared to *Pf* or *Pv* mono-infections. | McKenzie *et al.*, 2006 (18) |
| *Pf-Pv* | PNG | 1997-2004 | microscopy | *Pv* mono-infections and *Pf-Pv* co-infections are present in severe malaria without indication of protective effects. | Genton *et al.*, 2008 (19) |
| *Pf-Pv* | PNG | 2006 | PCR | *Pf-Pv* co-infections are common without indication that *Pv* is protective against *Pf* infections. | Colborn *et al.*, 2018 (20) |

* Meta-analysis on 73 datasets; † Retrospective analysis of 19 studies; ^‡^ Meta-analysis on 62 datasets

**Table B. Interaction between gametocytes of different Plasmodium species.**

| ***Plasmodium* species** | **Area of study** | **Year of study** | **Diagnostic method** | **Findings** | **Reference** |
| --- | --- | --- | --- | --- | --- |
| *Pf-Pm** | USA | 1940-1958 | microscopy | Increased *Pf* gametocyte density in neurosyphilis patients with *Pf-Pm* co-infections compared to *Pf* mono-infections. | McKenzie *et al.,* 2002 (21) |
| *Pf-Pm* | Kenya | 2004 | QT-NASBA | Increased *Pf* gametocyte density in *Pf-Pm* co-infections compared to *Pf* mono-infections. | Bousema *et al.,* 2008 (22) |
| *Pf-Pm* | Burkina Faso | 2007-2010 | microscopy | Reduced *Pf* gametocyte prevalence and density in *Pf-Pm* co-infections compared to *Pf* mono-infections. | Gnémé *et al.,* 2013 (23) |
| *Pf-Pv* | PNG | 2009-2010 | RT-qPCR | Reduced *Pf* and *Pv* gametocyte density in *Pf-Pv* co-infections compared to mono-infections. | Koepfli *et al.,* 2015 (24)  Wampfler *et al.,* 2017 (25) |
| *Pf-Pv* | Thailand | 1990-1995 | microscopy | Reduced *Pf* gametocyte prevalence in *Pf-Pv* co-infections compared to *Pf* mono-infections. | Price *et al.,* 1999 (26) |

* Malariotherapy in neurosyphilis patients (retrospective analysis)

1. **Assay conditions for *P. malariae* and *P. ovale* gametocyte-specific RT-qPCR targeting *pms25/pos25*.**

**Nucleic acid extractions**

For DNA or RNA extraction, finger-prick blood was sampled and stored as previously described^20,25^. Briefly, DNA was extracted from 100-150µl blood pellet using FavorPrep 96-well genomic DNA extraction kit (Favorgen, Taiwan). For RNA extraction, 50µl whole blood from each participant was spotted on a Whatman filter paper, air-dried, and after 2-4 weeks transferred into TRIzol reagent (Life Technologies, Switzerland) for long-term storage and transport. From all qPCR positive samples, RNA was extracted using RNeasy Plus Mini RNA kit (Qiagen, Switzerland) as described previously^20^. RNA was eluted in 50µl RNase-free dH2O.

**Study site and population**

Nucleic acid samples were collected from the Albinama cohort study conducted between 2009 and 2010 in villages of Maprik District, East Sepik Province, PNG. Details of the study design were published previously. In brief, after screening for G6PD deficiency, 504 children aged 5-10 years were recruited and randomized to two treatment arms for receiving directly observed treatment (DOT) over 4 weeks of either (i) chloroquine (CQ) (DOTs 1–3), artemether-lumefantrine (AL) (DOTs 11–13), and primaquine (PQ) (DOTs 1–20; 0.5 mg/kg); or (ii) CQ (DOTs 1–3), AL (DOTs 11–13), and placebo (PL) (DOTs 1–20). A high total dose of PQ in treatment arm (i) was used to effectively eliminate *Pv* and *Po* hypnozoites from the liver. A venous blood sample was collected three days after the final DOT, representing day 0 of follow-up (i.e. pre-treatment). Children were actively monitored for infection by qPCR and illness for a total of 32 weeks. Finger-prick blood samples were taken every two weeks for the first 12 weeks and every 4 weeks from week 14-32. Symptomatic children (i.e. fever and confirmed parasitemia by rapid diagnostic test) received treatment with AL over a 3-day course. *Pv* predominated with 48% of study participants positive by qPCR before treatment, followed by *Pf* (24%), *Pm* (15%) and *Po* (3%).

**Table C: Gametocyte-specific *pms25* and *pos25* RT-qPCR**

| **Species** | **Primer** | **Sequence (5’- 3’)** |
| --- | --- | --- |
| *P. malariae* | pms25_fw | 5’ – GTA AAA GAT GTT TGT GTT CCG TCT G – 3’ |
|  | pms25_rv | 5’ – CAC ATG TTT TTG TTA TTT GGA TCT GG – 3’ |
|  | pms25_probe | 5’ – **HEX** - CCA AAT AAT GAA GAT GTT AAA ACT GCC – **BHQ1** – 3’ |
| *P. ovale* | pos25_fw | 5’ – CGT ACC CGC TGA ATG CAA AGG – 3’ |
|  | pos25_rv | 5’ – GCC TAT ATT ACA TGA GCA TCT ACC – 3’ |
|  | pos25_probe | 5’ – **FAM** - AAC CCA AGC CCG GAT AAT – **MGB** – EQ – 3’ |

**Table D: RT-qPCR Reaction Mix** (Luna Universal Probe One-Step RT-qPCR Kit)

| **Component** | **Final concentration** | **Quantity per reaction** |
| --- | --- | --- |
| Luna Universal One-Step Reaction Mix 2X (NEB, catalog# E3006S) | 1X | 7µl |
| Luna WarmStart RT Enzyme Mix 20X | 1X | 0.7 µl |
| Primer mix fw & rv (10µM each) | 785 nM each | 1.1 µl |
| Probe (10µM) | 250 nM | 0.35 µl |
| Template |  | 4 µl |
| ddH_2_O |  | 0.85 µl |
| Total |  | 14 µl |

**Table E: RT-qPCR cycling parameters***

| **Stage** | **Temperature** | **Time (min)** | **Cycles** |
| --- | --- | --- | --- |
| Reverse Transcription | 55°C | 10:00 | 1 |
| Initial Denaturation | 95°C | 1:00 | 1 |
| Denaturation & Extension | 95°C | 0:10 | 45 |
|  | **58°C** | 1:00 |  |

*Platform: StepOne Plus Real-Time PCR System (Applied Biosystems)

**Performance *pms25* and *pos25* RT-qPCR**

For standard curves and internal controls, a control plasmid containing the *P. malariae pms25* or *P. ovale pos25* amplicon, respectively, was generated. The *pms25* and *pos25* PCR products were amplified from *P. malariae* or *P. ovale* genomic DNA, respectively, and cloned into the pCR 2.1- TOPO TA vector (Invitrogen). Analytical sensitivity and qPCR efficiency were determined on a plasmid dilution row in the range from 10^7^-0.01 copy numbers/µL. The performance parameters of the *pms25* and *pos25* RT-qPCR assays are shown in **Table F**.

**Table F: Performance parameters for *P. malariae* *pms25* RT-qPCR and *P. ovale* *pos25* RT-qPCR**

| **Target gene** | **Assay** | **Slope** | **Efficiency*** | **Intercept** | **R^2^** | **Amplicon size** | **LOD**  **Plasmid copies/µL DNA solution**** |
| --- | --- | --- | --- | --- | --- | --- | --- |
| *pms25* | TaqMan | -3.34 | 99.35% | 38.653 | 0.99 | 148 bp | 2 |
| *pos25* | TaqMan | -3.54 | 91.66% | 38.453 | 0.99 | 100 bp | 1 |

*considering plasmid concentrations of 10^7^-10 copies/µL. Applied threshold 0.04 for *pms25* and 0.1 for *pos25*, respectively.

**LOD was defined by the lowest concentration of the standard plasmid whereby at least 50% of the replicates were positive.

**Limit of Detection of *pms25* and *pos25* RT-qPCR**

**Table G: Serial dilution of *pms25* plasmid**

| **Plasmid** | ***pms25*** | |
| --- | --- | --- |
| **Copies/µL DNA solution*** | **Positivity** | **Mean C_t_ (±StDev)** |
| 10^7^ | 3/3 | 15.39±0.03 |
| 10^6^ | 3/3 | 18.44±0.26 |
| 10^5^ | 3/3 | 22.05±0.19 |
| 10^4^ | 3/3 | 25.34±0.53 |
| 10^3^ | 3/3 | 28.61±0.03 |
| 10^2^ | 3/3 | 31.91±0.20 |
| 10^1^ | 3/3 | 35.38±0.98 |
| 5 | 3/5 | 37.20±1.21 |
| 2 | 3/5 | 38.43±1.43 |
| 1 | 2/5 | 38.13±0.42 |
| 0.1 | 0/5 | n. a. |
| 0.01 | 0/5 | n. a. |

*4µL template DNA were added per PCR reaction

**Table H: Serial dilution of *pos25* plasmid**

| **Plasmid** | ***pos25*** | |
| --- | --- | --- |
| **Copies/µL DNA solution*** | **Positivity** | **Mean C_t_ (±StDev)** |
| 10^7^ | 3/3 | 13.87±0.07 |
| 10^6^ | 3/3 | 17.28±0.14 |
| 10^5^ | 3/3 | 20.33±0.07 |
| 10^4^ | 3/3 | 24.30±0.17 |
| 10^3^ | 3/3 | 27.77±0.07 |
| 10^2^ | 3/3 | 31.70±0.20 |
| 10^1^ | 3/3 | 34.81±0.68 |
| 5 | 5/5 | 35.29±0.30 |
| 2 | 4/5 | 36.05±0.67 |
| 1 | 5/5 | 36.65±0.12 |
| 0.1 | 2/5 | 39.82±1.66 |
| 0.01 | 1/5 | 42.84 |

*4µL template DNA were added per PCR reaction

**Intra-assay coefficient of variation (CV) of *pms25* and *pos25* RT-qPCR**

The intra-assay CV refers to the short-term precision or repeatability. Intra-assay variation of the *pms25* and *pos25* RT-qPCR assays were assessed by calculating coefficient of variation (CV, expressed in %) on the basis of the Ct-values of the amplification curves using three serially diluted plasmid standards (10^6^, 10^4^ and 10^2^ copies/µl) in triplicates (i.e., standard deviation divided by mean, and multiplied by 100).

**Table J: Intra-assay CV of *pms25* and *pos25* RT-qPCR**

|  | **copies/µl** | ***%CV (mean)** | **%CV IQR** |
| --- | --- | --- | --- |
| *pms25* assay | 10^6^ | **0.299** | 0.073 - 0.65 |
|  | 10^4^ | **0.336** | 0.046 - 0.711 |
|  | 10^2^ | **0.422** | 0.09 - 0.802 |
| *pos25* assay | 10^6^ | **0.935** | 0.661 - 1.172 |
|  | 10^4^ | **0.245** | 0.121 - 0.935 |
|  | 10^2^ | **0.496** | 0.323 - 0.651 |

*%CV (mean) = mean of all single %CV calculated

**Inter-assay coefficient of variation (CV)** **of *pms25* and *pos25* RT-qPCR**

The inter-assay CV refers to the long-term precision or reproducibility. Plasmid standard dilution rows were also used to assess inter-assay variation defined as the variation of Ct-values of three serially diluted plasmid standards (10^6^, 10^4^ and 10^2^ copies/µl) in triplicates of six independent RT-qPCR runs. The plate means were calculated and then used to calculate the overall mean, standard deviation (SD), and %CV

**Table K: Inter-assay CV of *pms25* and *pos25* RT-qPCR**

|  | **copies/µl** | **mean of means (Ct)** | **SD** | ***%CV (95%CI)** |
| --- | --- | --- | --- | --- |
| *pms25* assay | 10^6^ | 17.447 | 0.052 | **0.297** (± 0.042) |
|  | 10^4^ | 23.840 | 0.080 | **0.334** (± 0.07) |
|  | 10^2^ | 29.894 | 0.126 | **0.422** (± 0.11) |
| *pos25* assay | 10^6^ | 16.449 | 0.153 | **0.933** (± 0.12) |
|  | 10^4^ | 22.714 | 0.125 | **0.550** (± 0.1) |
|  | 10^2^ | 29.142 | 0.144 | **0.495** (± 0.12) |

*Overall % CV = SD of plate means ÷ mean of plate means x 100.

For both, inter- and intra CV a value ≤1 in qPCR indicates reproducible and repeatable result.

1. **Additional epidemiological analysis of co-infection in pre-treatment samples**

**Table L: Determinants of asexual parasite and gametocyte infection in pre-treatment samples.** Odds ratios with 95% confidence intervals were calculated using logistic regression. For the reference village of Albinama, the estimated model intercept is presented and not the odds ratio.

|  | ***P. falciparum* asexuals (24.0% = 121/505)** | | ***P. vivax* asexuals  (47.3% = 239/505)** | | ***P. malariae* asexuals  (14.3% = 72/505)** | | ***P. ovale* asexuals  (3.4% = 17/505)** | |
| --- | --- | --- | --- | --- | --- | --- | --- | --- |
|  | **odds ratio** | **P value** | **odds ratio** | **P value** | **odds ratio** | **P value** | **odds ratio** | **P value** |
| village: Albinama (intercept) | 0.134 (0.027, 0.665) |  | 1.824 (0.506, 6.574) |  | 0.065 (0.011, 0.394) |  | 0.046 (0.002, 1.067) |  |
| village: Amahup | 0.560 (0.274, 1.142) | 0.11 | 0.515 (0.309, 0.860) | 0.011 | 1.085 (0.446, 2.642) | 0.86 | 0.000 (0.000, Inf) | 0.99 |
| village: Balanga | 1.770 (0.836, 3.743) | 0.14 | 0.611 (0.318, 1.173) | 0.14 | 1.935 (0.729, 5.138) | 0.185 | 0.671 (0.130, 3.460) | 0.63 |
| village: Balif | 0.640 (0.321, 1.277) | 0.21 | 0.605 (0.364, 1.005) | 0.0522 | 0.947 (0.384, 2.336) | 0.91 | 0.000 (0.000, Inf) | 0.99 |
| village: Bolumita | 9.685 (4.80, 19.53) | 2.2 x 10^-10^ | 1.784 (0.951, 3.346) | 0.071 | 6.862 (3.003, 15.684) | 4.9 x 10^-6^ | 2.424 (0.788, 7.458) | 0.12 |
| age (in years) | 1.069 (0.921, 1.240) | 0.38 | 0.930 (0.828, 1.046) | 0.23 | 1.177 (0.992, 1.398) | 0.062 | 1.070 (0.783, 1.461) | 0.67 |
| sex (male) | 1.163 (0.731, 1.850) | 0.52 | 0.957 (0.668, 1.373) | 0.81 | 0.589 (0.339, 1.022) | 0.06 | 0.858 (0.311, 2.365) | 0.77 |
| bed net | 0.973 (0.405, 2.340) | 0.95 | 1.152 (0.542, 2.451) | 0.71 | 0.501 (0.213, 1.179) | 0.11 | 0.758 (0.186, 3.094) | 0.70 |
|  | ***P. falciparum* gametocytes (13.3% = 67/505)** | | ***P. vivax* gametocytes  (28.1% = 142/505)** | | ***P. malariae* gametocytes (6.9% = 35/505)** | | ***P. ovale* gametocytes  (2.2% = 11/505)** | |
|  | **odds ratio** | **P value** | **odds ratio** | **P value** | **odds ratio** | **P value** | **odds ratio** | **P value** |
| village: Albinama (intercept) | 0.036 (0.005, 0.252) |  | 1.860 (0.460, 7.521) |  | 0.009 (0.000, 0.018) |  | 0.087 (0.002, 3.883) |  |
| village: Amahup | 0.721 (0.309, 1.683) | 0.45 | 0.556 (0.318, 0.971) | 0.039 | 0.796 (0.194, 3.273) | 0.75 | 0.000 (0.000, +Inf) | 0.99 |
| village: Balanga | 1.376 (0.531, 3.565) | 0.51 | 0.658 (0.323, 1.341) | 0.25 | 3.082 (0.826, 11.506) | 0.094 | 0.000 (0.000, +Inf) | 0.99 |
| village: Balif | 0.522 (0.208, 1.311) | 0.167 | 0.485 (0.274, 0.858) | 0.013 | 0.865 (0.211, 3.554) | 0.84 | 0.000 (0.000, +Inf) | 0.99 |
| village: Bolumita | 5.336 (2.451, 11.62) | 0.000025 | 0.920 (0.488, 1.753) | 0.80 | 10.023 (3.15, 31.86) | 0.0001 | 3.847 (0.926, 15.977) | 0.064 |
| age (in years) | 1.047 (0.881, 1.244) | 0.60 | 0.866 (0.761, 0.986) | 0.03 | 0.984 (0.783, 1.236) | 0.89 | 0.926 (0.627, 1.367) | 0.69 |
| sex (male) | 1.314 (0.759, 2.275) | 0.33 | 0.803 (0.540, 1.195) | 0.28 | 0.529 (0.246, 1.138) | 0.10 | 1.053 (0.298, 3.722) | 0.94 |
| bed net | 2.187 (0.735, 6.505) | 0.16 | 1.004 (0.446, 2.26) | 0.99 | 6.479 (0.809, 51.894) | 0.078 | 0.515 (0.115, 2.314) | 0.39 |

**Table M: Effect of co-infection on parasite density.** Enrolment (pre-treatment) samples only. Here, single infection indicates that no other parasite species is present, and co-infection indicates that at least one other parasite species is present.

|  | **single infection** | | **co-infection** | | **P value** |
| --- | --- | --- | --- | --- | --- |
|  | **copy number** | **N** | **copy number** | **N** |  |
| **asexual parasites** | | | | | |
| *P. falciparum* | 109.7 (5.7, 71724) | 36 | 146.4 (6.0, 14891.4) | 85 | 0.49 |
| *P. vivax* | 5.2 (0.0, 129.8) | 151 | 5.4 (0.0, 599.1) | 88 | 0.29 |
| *P. malariae* | 23.3 (0.4, 314.9) | 18 | 87.9 (1.4, 1210.8) | 54 | 0.0055 |
| *P. ovale* | 6.0 (6.0, 6.0) | 1 | 3.2 (3.2, 14988.5) | 16 | 0.75 |
| **gametocytes** | | | | | |
| *P. falciparum* | 10.8 (0.3, 1453.4) | 25 | 13.7 (0.2, 223.9) | 42 | 0.86 |
| *P. vivax* | 4.5 (0.1, 68.2) | 100 | 3.6 (0.1, 273.7) | 42 | 0.68 |
| *P. malariae* | 0.16 (0.08, 0.70) | 4 | 1.45 (0.06, 94.9) | 31 | 0.086 |
| *P. ovale* |  | 0 | 1.18 (0.08, 13.3) | 11 | 1 |

**Table N: Plasmodium asexual and gametocyte infection status in 504 enrolment samples.**

| *Plasmodium* species | N positive* | % gametocyte positive (n/N)† | % gametocyte negative (n/N) † | % gametocyte positive in mono-infections† | % gametocyte positive in co-infections†,^‡^ |
| --- | --- | --- | --- | --- | --- |
| *P. falciparum* | 121 | 55.4 (67/121) | 44.6 (54/121) | 55.2 (37/67) | 44.7 (30/67) |
| *P. vivax* | 239 | 59.4 (142/239) | 40.6 (97/239) | 77.5 (110/142) | 22.5 (32/142) |
| *P. malariae* | 72 | 48.6 (35/72) | 51.4 (37/72) | 40 (14/35) | 60 (21/35) |
| *P. ovale* | 17 | 64.7 (11/17) | 35.3 (6/17) | 9.1 (1/11) | 90.9 (10/11) |

*determined by 18S rRNA qPCR

†determined by gametocyte-specific RT-qPCR

^‡^double, triple and quadruple co-infections

1. **Epidemiological analysis of co-infection in samples from the entire cohort**

**Table O: Determinants of asexual parasite co-infection in all samples.** Odds ratios with 95% confidence intervals were calculated using logistic regression. For the reference village of Albinama, the estimated model intercept is presented and not the odds ratio.

|  | ***P. falciparum* asexuals (9.9% = 554/5561)** | | ***P. vivax* asexuals  (17.2% = 955/5561)** | | ***P. malariae* asexuals  (2.2% = 123/5561)** | | ***P. ovale* asexuals  (0.9% = 52/5561)** | |
| --- | --- | --- | --- | --- | --- | --- | --- | --- |
|  | **odds ratio** | **P value** | **odds ratio** | **P value** | **odds ratio** | **P value** | **odds ratio** | **P value** |
| village: Albinama (intercept) | 0.061 (0.032, 0.115) |  | 0.892 (0.539, 1.476) |  | 0.006 (0.002, 0.023) |  | 0.006 (0.001, 0.04) |  |
| village: Amahup | 0.506 (0.349, 0.735) | 0.00034 | 0.359 (0.282, 0.456) | < 2.0 x 10^-16^ | 0.842 (0.435, 1.630) | 0.61 | 0.442 (0.129, 1.517) | 0.19 |
| village: Balanga | 1.770 (1.255, 2.496) | 0.0011 | 1.158 (0.911, 1.474) | 0.23 | 2.282 (1.210, 4.304) | 0.011 | 1.723 (0.599, 4.961) | 0.31 |
| village: Balif | 0.981 (0.716, 1.344) | 0.91 | 0.651 (0.527, 0.803) | 0.000063 | 0.654 (0.328, 1.304) | 0.23 | 0.663 (0.222, 1.980) | 0.46 |
| village: Bolumita | 8.094 (6.08, 10.77) | < 2.0 x 10^-16^ | 2.177 (1.745, 2.715) | 5.2 x 10^-12^ | 4.557 (2.595, 9.003) | 1.3 x 10^-7^ | 6.816 (2.919, 15.920) | 9.2 x 10^-6^ |
| age (in years) | 1.058 (0.997, 1.123) | 0.062 | 0.921 (0.879, 0.967) | 0.00079 | 1.175 (1.047, 1.318) | 0.0062 | 1.009 (0.848, 1.200) | 0.918 |
| sex (male) | 0.855 (0.707, 1.035) | 0.11 | 0.895 (0.772, 1.038) | 0.14 | 1.085 (0.751, 1.567) | 0.66 | 0.830 (0.471, 1.462) | 0.52 |
| bed net | 0.836 (0.623, 1.121) | 0.23 | 0.800 (0.617, 1.039) | 0.255 | 0.704 (0.412, 1.202) | 0.20 | 1.300 (0.529, 3.193) | 0.57 |
| treatment (primaquine) | 0.825 (0.683, 0.997) | 0.047 | 0.360 (0.308, 0.420) | < 2.0 x 10^-16^ | 0.776 (0.538, 1.120) | 0.18 | 0.620 (0.353, 1.091) | 0.097 |
|  | ***P. falciparum* gametocytes (3.2% = 179/5561)** | | ***P. vivax* gametocytes  (7.5% = 416/5561)** | | ***P. malariae* gametocytes (0.8% = 43/5561)** | | ***P. ovale* gametocytes  (0.3% = 17/5561)** | |
|  | **odds ratio** | **P value** | **odds ratio** | **P value** | **odds ratio** | **P value** | **odds ratio** | **P value** |
| village: Albinama (intercept) | 0.010 (0.004, 0.029) |  | 0.674 (0.337, 1.348) |  | 0.002 (0.000, 0.018) |  | 0.005 (0.000, 0.109) |  |
| village: Amahup | 0.609 (0.329, 1.129) | 0.12 | 0.401 (0.290, 0.554) | 3.0 x 10^-8^ | 0.647 (0.197, 2.130) | 0.47 | 0.000 (0.000, +Inf) | 0.99 |
| village: Balanga | 1.567 (0.857, 2.865) | 0.14 | 0.901 (0.645, 1.259) | 0.54 | 2.089 (0.695, 6.275) | 0.19 | 0.458 (0.051, 4.131) | 0.47 |
| village: Balif | 1.005 (0.586, 1.722) | 0.98 | 0.538 (0.400, 0.723) | 0.000039 | 0.516 (0.145, 1.832) | 0.31 | 0.191 (0.021, 1.713) | 0.14 |
| village: Bolumita | 6.315 (3.93, 10.136) | 2.2 x 10^-14^ | 1.283 (0.951, 1.732) | 0.10 | 6.182 (2.452, 15.583) | 0.0001 | 4.084 (1.251, 13.337) | 0.019 |
| age (in years) | 1.086 (0.988, 1.194) | 0.088 | 0.869 (0.812, 0.929) | 0.000045 | 1.033 (0.856, 1.247) | 0.73 | 0.957 (0.710, 1.290) | 0.77 |
| sex (male) | 1.099 (0.808, 1.496) | 0.55 | 0.803 (0.653, 0.986) | 0.036 | 0.875 (0.472, 1.621) | 0.67 | 0.840 (0.317, 2.221) | 0.72 |
| bed net | 1.019 (0.638, 1.628) | 0.94 | 0.686 (0.484, 0.972) | 0.033 | 2.205 (0.654, 7.440) | 0.20 | 0.925 (0.250, 3.426) | 0.91 |
| treatment (primaquine) | 1.020 (0.751, 1.384) | 0.90 | 0.482 (0.390, 0.597) | 2.0 x 10^-11^ | 1.011 (0.551, 1.857) | 0.97 | 1.236 (0.465, 3.290) | 0.67 |

**Table P: Estimated risk of co-infection in all samples.** *corrected for heterogeneity in exposure.

|  | **Asexual parasites** | | **Gametocytes** | |
| --- | --- | --- | --- | --- |
|  | risk | P value | risk | P value |
| ***All samples*** |  |  |  |  |
| excess risk of 2^nd^ infection | 2.43 (2.10, 2.76) | 0.00037 | 2.43 ( 1.31, 3.55) | 0.055 |
| excess risk of 3^rd^ infection | 14.00 (0.57, 27.45) | 0.15 | 63.66 ( -8.01, 135.32) | 0.19 |
| heterogeneity adjusted excess risk of 3^rd^ infection * | 2.37 (0.10, 4.64) | 0.32 | 10.78 (-1.36, 22.92) | 0.21 |
| ***All samples; stratified by village*** | | | | |
| excess risk of 2^nd^ infection | 1.49 (1.42, 1.57) | 3.3 x 10^-14^ | 1.76 (1.50, 2.02) | 1.8 x 10^-6^ |
| excess risk of 3^rd^ infection | 4.26 (3.89, 4.64) | 1.7 x 10^-14^ | 21.02 (19.12, 22.92) | 2.4 x 10^-16^ |
| heterogeneity adjusted excess risk of 3^rd^ infection * | 1.91 (1.74, 2.08) | 2.7 x 10^-10^ | 6.78 (6.17, 7.39) | 2.7 x 10^-15^ |

**Table Q: Effect of co-infection on parasite density.** All samples from the cohort. Here, single infection indicates that no other parasite species is present, and co-infection indicates that at least one other parasite species is present.

|  | **single infection** | | **co-infection** | | **P value** |
| --- | --- | --- | --- | --- | --- |
|  | **copy number** | **N** | **copy number** | **N** |  |
| **asexual parasites** | | | | | |
| *P. falciparum* | 567.1 (5.7, 190153) | 300 | 159.5 (4.0, 62012) | 259 | 5.7 x 10^-6^ |
| *P. vivax* | 11.6 (0.02, 297.8) | 697 | 10.6 (0.06, 615.2) | 265 | 0.86 |
| *P. malariae* | 42.1 (0.4, 820.2) | 39 | 44.4 (2.3, 1147.0) | 84 | 0.35 |
| *P. ovale* | 6.5 (0.3, 196.0) | 11 | 3.2 (0.2, 136.0) | 41 | 0.19 |
| **gametocytes** | | | | | |
| *P. falciparum* | 4.0 (0.2, 563.2) | 113 | 6.3 (0.2, 305.8) | 67 | 0.29 |
| *P. vivax* | 1.74 (0.02, 60.54) | 325 | 1.59 (0.06, 40.45) | 91 | 0.43 |
| *P. malariae* | 0.10 (0.05, 0.67) | 6 | 1.06 (0.02, 76.2) | 37 | 0.035 |
| *P. ovale* | 0.14 (0.01, 0.27) | 2 | 0.64 (0.04, 13.2) | 15 | 0.12 |


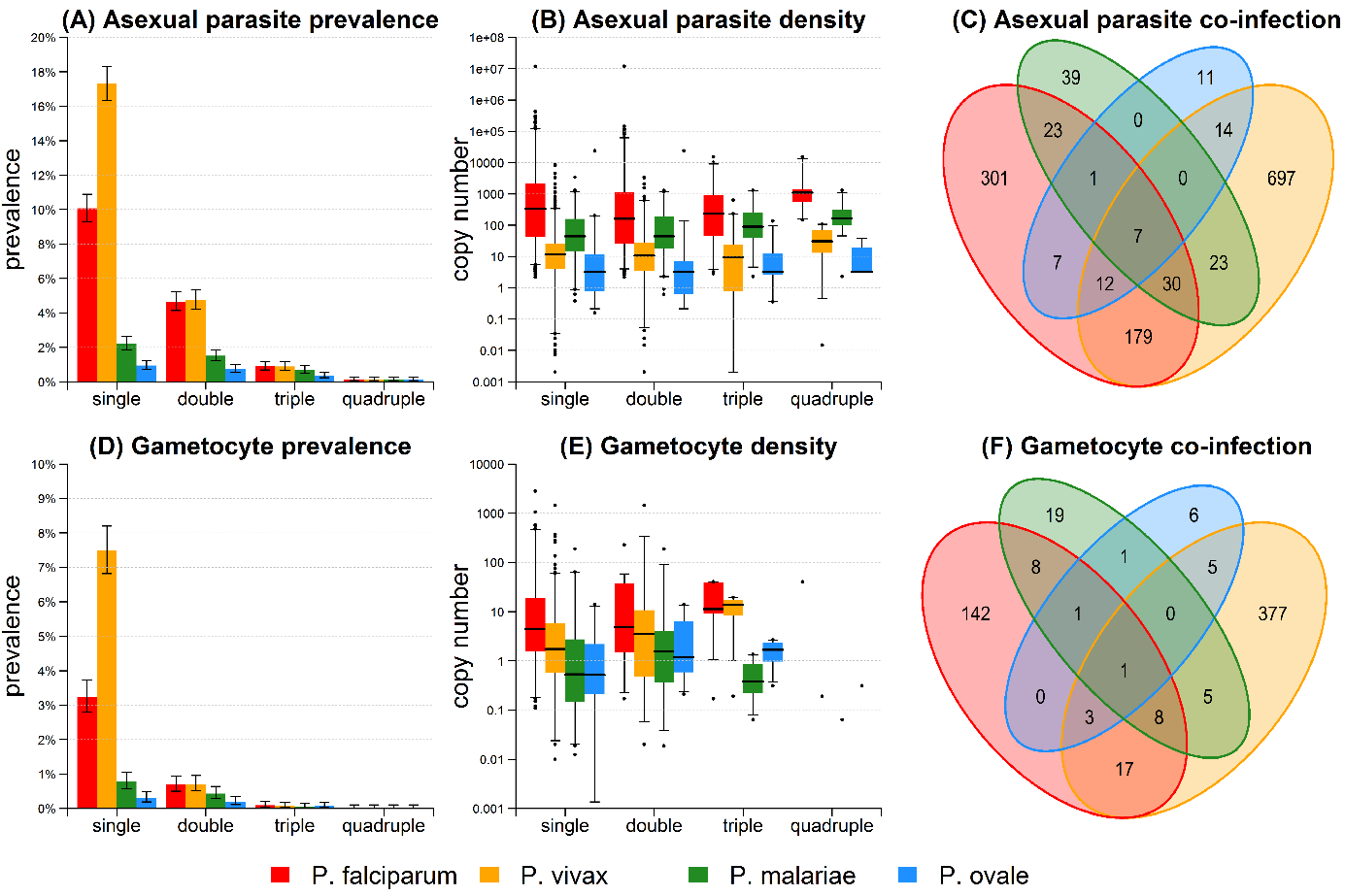


**Figure B: Malaria co-infection in all samples (n = 5561). (A)**  Co-infection prevalence of asexual parasites. Double infection with Pf denotes the proportion of samples PCR positive for Pf and at least one other species. Triple infection with Pf denotes the proportion of samples PCR positive for Pf and at least two other species. Other bars are similarly defined. **(B)** Asexual parasite density in co-infected samples. **(C)** Venn diagram of asexual parasite co-infection. **(D)**  Co-infection prevalence of gametocytes. **(E)** Gametocyte density in co-infected samples. **(F)** Venn diagram of gametocyte co-infection.


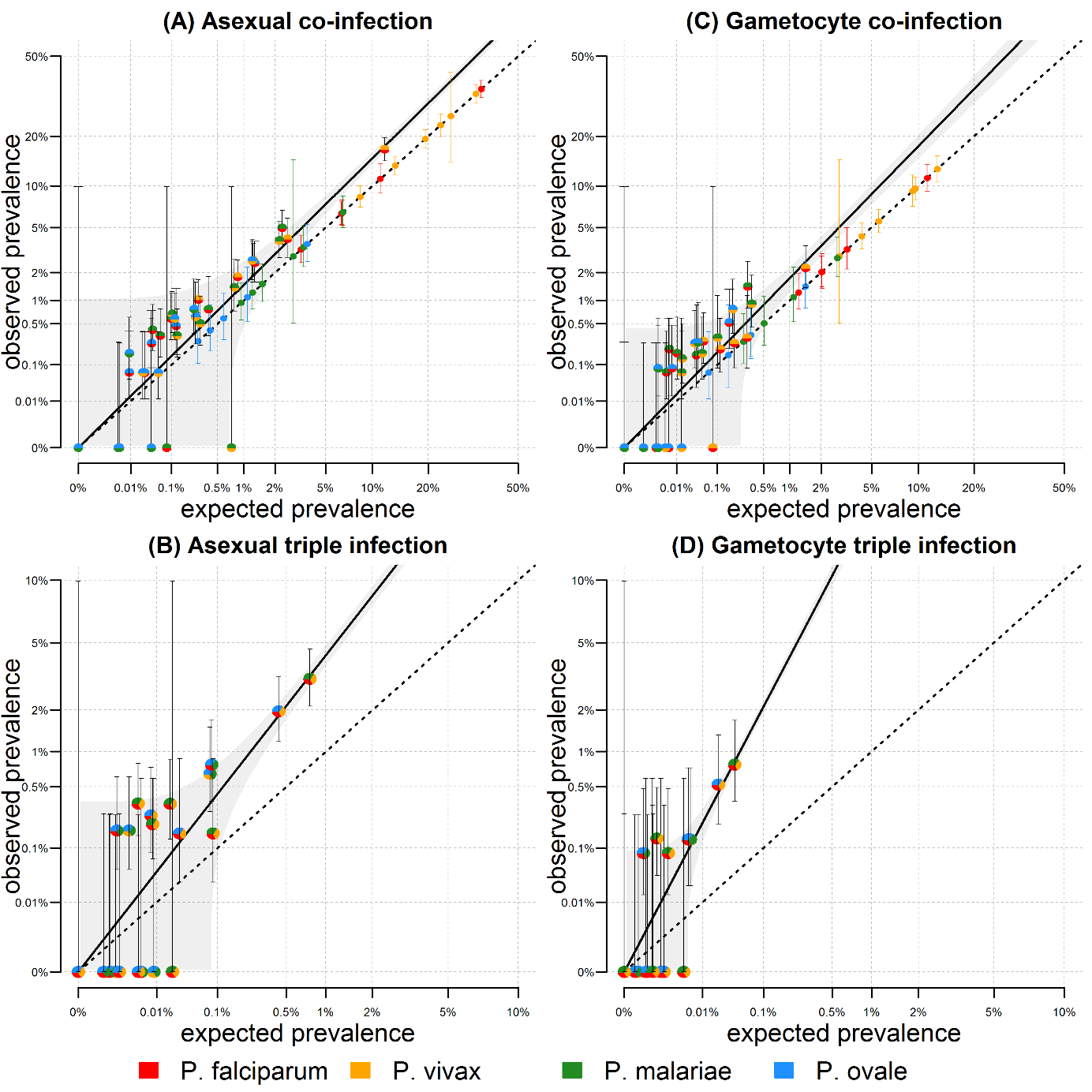


**Figure C: Co-infection in all samples stratified by village. (A)** For the six pairwise combinations of two malaria species, the observed and expected co-infection prevalence is plotted for each of the five villages. If the prevalence of Pf is X_Pf_ and the prevalence of Pv is X_Pv_, then we assume that the expected co-infection prevalence of Pf and Pv is X_Pf_ * X_Pv_. The dashed line represents the scenario where observed co-infection prevalence equals expected prevalence. The multi-coloured data points tend to fall above this line. The solid line denotes a regression model fitted through these points, with 95% confidence intervals shown in grey. **(B)** Observed and expected triple infection. If the prevalence of Pm is X_Pm_, then the expected prevalence of Pf, Pv and Pm co-infection is X_Pf_ * X_Pv_ * X_Pm_. The multi-coloured points fall above the dashed line indicating greater observed than expected prevalence. The solid line denotes a regression model fitted through these points. **(C)** Gametocyte co-infection. **(D)** Gametocyte triple infection.


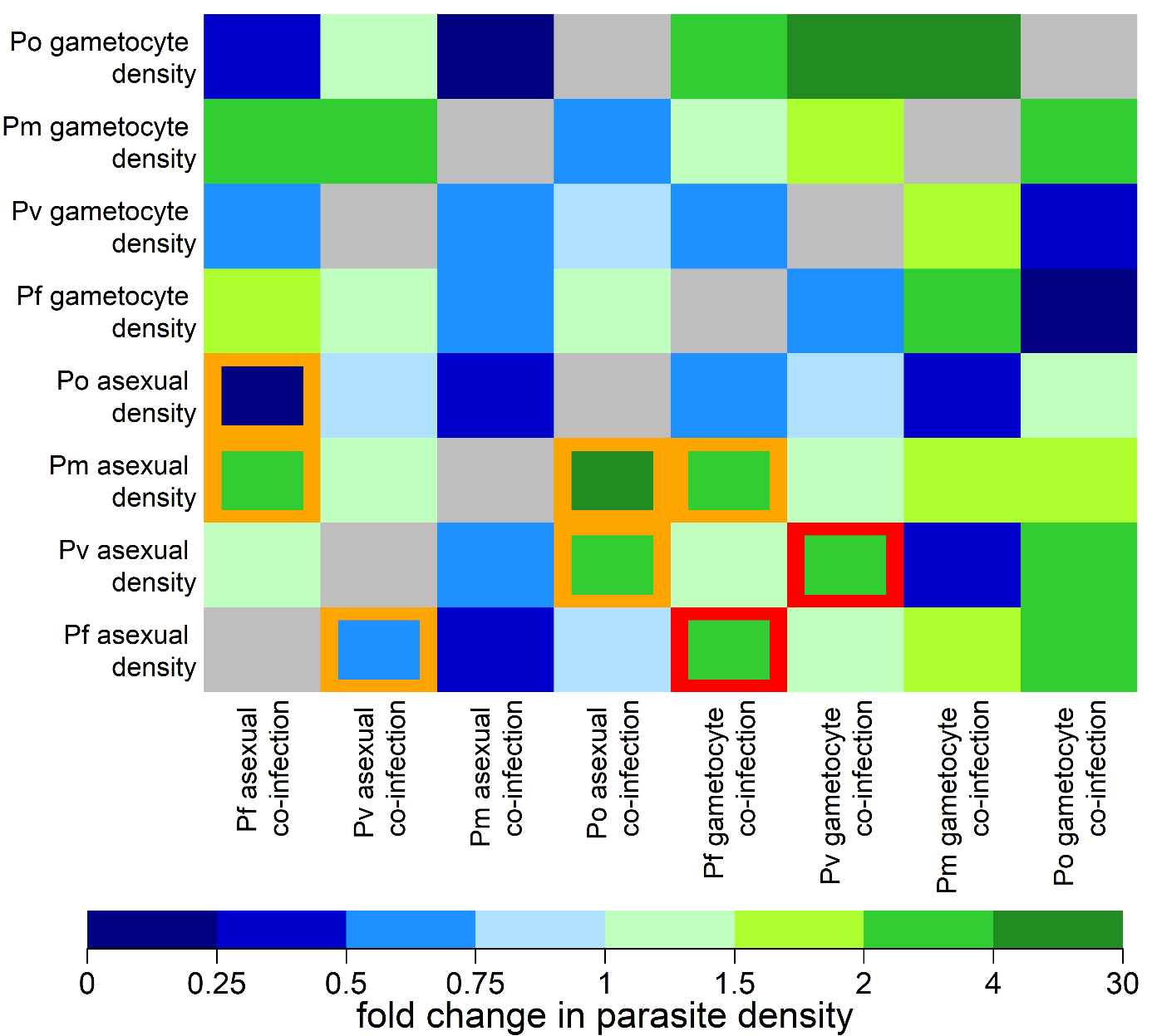


**Figure D: Effect of co-infection on parasite density in all samples.** Each square denotes the fold change in parasite density due to co-infection. For example, for Pm asexual parasites, co-infection with Pf asexual parasites leads to a 2.61 (1.4, 5.0) fold increase in Pm asexual parasite density. However, this was not significant after correction for multiple hypothesis testing (P = 0.08). Orange squares denote associations with P values < 0.05. Red squares denote association with P values < 0.05 after the Benjamini-Hochberg adjustment for multiple hypothesis testing.

1. **References**
2. Robinson LJ, Wampfler R, Betuela I, et al., Strategies for understanding and reducing the Plasmodium vivax and Plasmodium ovale hypnozoite reservoir in Papua New Guinean children: A randomised placebo-controlled trial and mathematical model. PLOS Med. 2015; 12, e1001891.
3. Howard SC, Donnelly CA, Chan MS. Methods for estimation of associations between multiple species parasite infections. *Parasitology*. 2001; **122**, 233–251.
4. Browne EN, *et al*, Malariometric update for the rainforest and savanna of Ashanti region, Ghana. *Ann. Trop. Med. Parasitol.* 2000; **94**, 15–22.
5. Molineaux L, Storey J, Cohen JE, Thomas A. A longitudinal study of human malaria in the West African Savanna in the absence of control measures: relationships between different Plasmodium species, in particular *P. falciparum* and *P. malariae*. *Am. J. Trop. Med. Hyg.* 1980; **29**, 725–737.
6. McKenzie FE, Bossert WH. Multispecies *Plasmodium* infections of humans. *J. Parasitol.* 1999; **85**, 12–18.
7. Marques PX, *et al.*, *Plasmodium* species mixed infections in two areas of Manhiça District, Mozambique. *Int. J. Biol. Sci.* 2005; 96–102.
8. Doctor SM, *et al.* Low prevalence of *Plasmodium malariae* and *Plasmodium ovale* mono-infections among children in the Democratic Republic of the Congo: a population-based, cross-sectional study. *Malar. J.* 2016; **15**, 350.
9. Woldearegai TG, *et al.* Characterization of *Plasmodium* infections among inhabitants of rural areas in Gabon. *Sci Rep.* 2019; **9**, 9784.
10. Black J, Hommel M, Snounou G, Pinder M. Mixed infections with *Plasmodium falciparum* and *P. malariae* and fever in malaria. *Lancet*. 1994; **343**, 1095.
11. Smith T, *et al.* Prospective risk of morbidity in relation to malaria infection in an area of high endemicity of multiple species of *Plasmodium*. *Am. J. Trop. Med. Hyg.* 2001; **64**, 262–267.
12. Haghdoost AA, Alexander N. Systematic review and meta-analysis of the interaction between *Plasmodium falciparum* and *Plasmodium vivax* in humans. *J. Vector Borne Dis.* 2007; **44**, 33–43.
13. Williams TN, *et al.*, High incidence of malaria in α-thalassaemic children. *Nature*. 1996; **383**, 522–525.
14. Mockenhaupt FP, Rong B, Till H, Thompson WN, Bienzle U. Short report: increased susceptibility to *Plasmodium malariae* in pregnant alpha(+)-thalassemic women. *Am. J. Trop. Med. Hyg.* 2001; **64**, 6–8.
15. Akala HM, *et al.* *Plasmodium* interspecies interactions during a period of increasing prevalence of *Plasmodium ovale* in symptomatic individuals seeking treatment: an observational study. *Lancet Microbe.* 2021; https:/doi.org/10.1016/S2666-5247(21)00009-4.
16. Luxemburger C, *et al.* The epidemiology of severe malaria in an area of low transmission in Thailand. *Trans. Roy. Soc. Trop. Med. Hyg.* 1997; **91**, 256–262.
17. Price RN, *et al.*, Factors contributing to anemia after uncomplicated *falciparum* malaria. *Am. J. Trop. Med. Hyg.* 2001; **65**, 614–622.
18. Price RN, *et al.*, Artesunate/mefloquine treatment of multi-drug resistant *falciparum* malaria. *Trans. Roy. Soc. Trop. Med. Hyg.* 1997; **91**, 574–577.
19. McKenzie FE, *et al.* Fever in patients with mixed-species malaria. *Clin. Infect. Dis.* 2006; **42**, 1713–1718.
20. Genton B, *et al.* *Plasmodium vivax* and mixed infections are associated with severe malaria in children: A prospective cohort study from Papua New Guinea. *PLOS Med.* 2008; **5**, e127.
21. Colborn KL, Mueller I, Speed TP. Joint modeling of mixed *Plasmodium* species infections using a bivariate Poisson lognormal model. *Am. J. Trop. Med. Hyg.* 2018; **98**, 71–76 (2018).
22. McKenzie FE, Jeffery GM, Collins WE. *Plasmodium malariae* infection boosts *Plasmodium falciparum* gametocyte production. *Am. J. Trop. Med. Hyg.* 2002; **67**, 411–414.
23. Bousema JT, *et al*. Increased *Plasmodium falciparum* gametocyte production in mixed infections with *P. malariae*. *Am J Trop Med Hyg.* 2008; 78, 442–448.
24. Gnémé A, *et al.* *Plasmodium* species occurrence, temporal distribution and interaction in a child-aged population in rural Burkina Faso. *Malar. J.* 2013; **12**, 67.
25. Koepfli C, *et al*. Blood-stage parasitaemia and age determine *Plasmodium falciparum* and *P. vivax* gametocytaemia in Papua New Guinea. *PLOS One*. 2015; 10, e0126747.
26. Wampfler R, *et al*. Effects of liver-stage clearance by primaquine on gametocyte carriage of *Plasmodium vivax* and *P. falciparum*. *PLOS Negl Trop Dis*. 2017; 11, e0005753.
27. Price R, *et al.* Risk factors for gametocyte carriage in uncomplicated falciparum malaria. *Am. J. Trop. Med. Hyg.* 1999; **60**, 1019–1023.
