# Supplementary material for: Co-infection of the four major Plasmodium species: Effects on densities and gametocyte carriage
Source: PLoS Negl Trop Dis. 2022 Sep 13;16(9):e0010760. doi: 10.1371/journal.pntd.0010760 (PMC9506632; doi:10.1371/journal.pntd.0010760)
Supplement: Attachment — Submitted filename: PNG_coinfections_editorial_notes.docx [file pntd.0010760.s002.docx]

**Senior Editorial Team**

**PLOS Neglected Tropical Diseases**

Paris, 6^th^ July 2022

Re: “**Co-infection of the four major Plasmodium species: effects on densities and gametocyte carriage**”

Following editorial comments from Anitha Samidurai, we would like to submit an updated version of our manuscript – “Co-infection of the four major Plasmodium species: effects on densities and gametocyte carriage” – for consideration for publication in PLOS Neglected Tropical Diseases.

With many thanks and kind regards,

Ingrid Felger	Michael White

*Professor em. Infection Biology, University of Basel	Infectious Disease Epidemiology & Analytics Unit*

*Swiss Tropical & Public Health Institute (Swiss TPH)	Department of Global Health*

*Dept. Medical Parasitology & Infection Biology	Institut Pasteur*

**Editorial comments**

1. Please ensure that the CRediT author contributions listed for every co-author are completed accurately and in full.

   At this stage, the following Authors require contributions: " All Authors ". Please ensure that the full contributions of each author are acknowledged in the ""Add/Edit/Remove Authors"" section of our submission form.

   The list of CRediT author contributions may be found here:

   https://urldefense.com/v3/__https://journals.plos.org/plosntds/s/authorship*loc-author-contributions__;Iw!!JFdNOqOXpB6UZW0!oe_XInJDePbyu1SH4bshtQoP2rAo5XYPUWZLFKgvCgvdw1_omibX5D4-sx7tvAzCdxAwHksLOKY1FFoAaTR9bYk$

   A new section on CRediT author contributions has been added (lines 542 – 553).
2. Please ensure that all author names are spelled correctly in the manuscript file, including the use of a middle name/initials, and any special characters (accents, umlauts, etc.). Please place first/given names before last/family names. All authors must have a first and last name. We noticed that Natalie E. Hofmann, Leanne J. Robinson are spelled differently between the manuscript file and submission form. Please be aware that correct spelling and formatting of author names in the article file is vital for accurate indexing in PubMed.

   All names have been checked and updated, both in the manuscript and the submission form. They are now all consistent.
3. We do not publish any copyright or trademark symbols that usually accompany proprietary names, eg (R), (C), or TM (e.g. next to drug or reagent names). Please remove all instances of trademark/copyright symbols throughout the text, including pCR® on Line number 198.

   All references to copyright or trademark symbols have been removed.
4. Please provide an Author Summary. This should appear in your manuscript between the Abstract (if applicable) and the Introduction, and should be 150–200 words long. The aim should be to make your findings accessible to a wide audience that includes both scientists and non-scientists. Sample summaries can be found on our website under Submission Guidelines:

   https://urldefense.com/v3/__https://journals.plos.org/plosntds/s/submission-guidelines*loc-parts-of-a-submission__;Iw!!JFdNOqOXpB6UZW0!oe_XInJDePbyu1SH4bshtQoP2rAo5XYPUWZLFKgvCgvdw1_omibX5D4-sx7tvAzCdxAwHksLOKY1FFoAXIuK2Xg$

   A new Author Summary has been added (lines 74 – 88).
5. Please ensure that the funders and grant numbers match between the Financial Disclosure field and the Funding Information tab in your submission form. Note that the funders must be provided in the same order in both places as well.
   Currently, Field work was supported by the TransEPI consortium funded by the Bill & Melinda Gates Foundation. IM (GNT11155075) and L.J.R. (GNT1161627) are supported by NHMRC Fellowships is missing from the Funding Information.

   The Funding Information tab has been updated so that it matches the statement in the manuscript.
6. Please amend your detailed Financial Disclosure statement. This is published with the article, therefore should be completed in full sentences and contain the exact wording you wish to be published.

   i). State the initials, alongside each funding source, of each author to receive each grant.

   The Funding statement has been separated out from the Acknowledgements into its own section (lines 485 – 494). All funding sources have been attributed to the specific individuals using their initials.
7. Please amend your detailed Financial Disclosure statement. This is published with the article, therefore should be completed in full sentences and contain the exact wording you wish to be published.

   i). State what role the funders took in the study. If the funders had no role in your study, please state: “The funders had no role in study design, data collection and analysis, decision to publish, or preparation of the manuscript.”

   ii). If any authors received a salary from any of your funders, please state which authors and which funders.

   The financial disclosure statement has now been updated to exactly match this statement (lines 250 – 251).
