# Supplementary material for: Co-infection of the four major Plasmodium species: Effects on densities and gametocyte carriage
Source: PLoS Negl Trop Dis. 2022 Sep 13;16(9):e0010760. doi: 10.1371/journal.pntd.0010760 (PMC9506632; doi:10.1371/journal.pntd.0010760)
Supplement: Attachment — Submitted filename: PNG_coinfections_editorial_notes_reviewer_responses.docx [file pntd.0010760.s003.docx]

**Senior Editorial Team**

**PLOS Neglected Tropical Diseases**

Paris, 19^th^ August 2022

Re: “**Co-infection of the four major Plasmodium species: effects on densities and gametocyte carriage**”

Following editorial comments from Anitha Samidurai, we would like to submit an updated version of our manuscript – “Co-infection of the four major Plasmodium species: effects on densities and gametocyte carriage” – for consideration for publication in PLOS Neglected Tropical Diseases. Please note that responses to different rounds of editorial and reviewers’ comments have been included below.

With many thanks and kind regards,

Ingrid Felger	Michael White

*Professor em. Infection Biology, University of Basel	Infectious Disease Epidemiology & Analytics Unit*

*Swiss Tropical & Public Health Institute (Swiss TPH)	Department of Global Health*

*Dept. Medical Parasitology & Infection Biology	Institut Pasteur*

**Editorial comments (August 2022)**

1. We notice that your revision was submitted on [AUG 19, 2022], but the manuscript file in your submission's file inventory was uploaded on [JULY 6,2022]. Please upload the latest version of your revised manuscript as the main article file, with the item type 'Manuscript,' ensuring that it does not contain any tracked changes or highlighting. This will be used in the production process if your manuscript is accepted.

An up to date version of the manuscript, figures and all other related material has been uploaded.

2. Please ensure that the CRediT author contributions listed for every co-author are completed accurately and in full. At this stage, "All Authors" require contributions. Please ensure that the full contributions of each author are acknowledged in the ""Add/Edit/Remove Authors"" section of our submission form.

The CRediT author contributions have now been updated on the Editorial Manager.

3. Please upload all main figures (Figure 4) as separate Figure files in .tif or .eps format.

All figures have been uploaded in .tif format.

4. We have noticed that you have uploaded Supporting Information file, but you have not included a list of legends. Please add a full list of legends for your Supporting Information files after the references list.

The Supporting Information files contains a substantial degree of additional information, totalling 4 figures and 16 tables. This information would be very useful for other researchers wishing to replicate our work, our implement these new assays in their own laboratories. Rather than providing 20 individual legends, we believe it is better to include the short description of the content of the Additional file as already provided.

5. We have noticed that you have included "Supplementary Table 7" twice in the uploaded Supplementary file.

This is an excellent observation. Supplementary Table 8 was mistakenly labelled as Supplementary Table 7. This has now been updated.

6. Thank you for including your ethics statement: " Human subjects: The study received ethical clearance from the PNG IMR Institutional Review Board (0908), the PNG Medical Advisory Committee (09.11), the Ethics Committee of Basel 237/11 and was conducted in full concordance with the Declaration of Helsinki. Written informed consent was obtained from the parents/guardians of all children enrolled in the study". Please state the full name of " PNG, IMR".

This has been updated as Papua New Guinea Institute of Medical Research.

7. Please amend your detailed Financial Disclosure statement. This is published with the article, therefore should be completed in full sentences and contain the exact wording you wish to be published.
   i. State what role the funders took in the study. If the funders had no role in your study, please state: “The funders had no role in study design, data collection and analysis, decision to publish, or preparation of the manuscript.”

This statement has been added to the funding section.

ii. If any authors received a salary from any of your funders, please state which authors and which funders.

The funders supported field and laboratory work. Salary for the authors was provided by their host institutions.

8. Please ensure that the funders and grant numbers match between the Financial Disclosure field and the Funding Information tab in your submission form. Note that the funders must be provided in the same order in both places as well. Currently, order of grants are different in both places.

The order of the grants has been updated so that it is the same in the funding section of the manuscript and the online portal.

9. We note that your "Revised article" files are duplicated on your submission. Please remove any unnecessary or old files from your revision, and make sure that only those relevant to the current version of the manuscript are included.

All duplicate files have been removed.

**Editorial comments (July 2022)**

1. Please ensure that the CRediT author contributions listed for every co-author are completed accurately and in full.

   At this stage, the following Authors require contributions: " All Authors ". Please ensure that the full contributions of each author are acknowledged in the ""Add/Edit/Remove Authors"" section of our submission form.

   The list of CRediT author contributions may be found here:

   https://urldefense.com/v3/__https://journals.plos.org/plosntds/s/authorship*loc-author-contributions__;Iw!!JFdNOqOXpB6UZW0!oe_XInJDePbyu1SH4bshtQoP2rAo5XYPUWZLFKgvCgvdw1_omibX5D4-sx7tvAzCdxAwHksLOKY1FFoAaTR9bYk$

   A new section on CRediT author contributions has been added (lines 542 – 553).
2. Please ensure that all author names are spelled correctly in the manuscript file, including the use of a middle name/initials, and any special characters (accents, umlauts, etc.). Please place first/given names before last/family names. All authors must have a first and last name. We noticed that Natalie E. Hofmann, Leanne J. Robinson are spelled differently between the manuscript file and submission form. Please be aware that correct spelling and formatting of author names in the article file is vital for accurate indexing in PubMed.

   All names have been checked and updated, both in the manuscript and the submission form. They are now all consistent.
3. We do not publish any copyright or trademark symbols that usually accompany proprietary names, eg (R), (C), or TM (e.g. next to drug or reagent names). Please remove all instances of trademark/copyright symbols throughout the text, including pCR® on Line number 198.

   All references to copyright or trademark symbols have been removed.
4. Please provide an Author Summary. This should appear in your manuscript between the Abstract (if applicable) and the Introduction, and should be 150–200 words long. The aim should be to make your findings accessible to a wide audience that includes both scientists and non-scientists. Sample summaries can be found on our website under Submission Guidelines:

   https://urldefense.com/v3/__https://journals.plos.org/plosntds/s/submission-guidelines*loc-parts-of-a-submission__;Iw!!JFdNOqOXpB6UZW0!oe_XInJDePbyu1SH4bshtQoP2rAo5XYPUWZLFKgvCgvdw1_omibX5D4-sx7tvAzCdxAwHksLOKY1FFoAXIuK2Xg$

   A new Author Summary has been added (lines 74 – 88).
5. Please ensure that the funders and grant numbers match between the Financial Disclosure field and the Funding Information tab in your submission form. Note that the funders must be provided in the same order in both places as well.
   Currently, Field work was supported by the TransEPI consortium funded by the Bill & Melinda Gates Foundation. IM (GNT11155075) and L.J.R. (GNT1161627) are supported by NHMRC Fellowships is missing from the Funding Information.

   The Funding Information tab has been updated so that it matches the statement in the manuscript.
6. Please amend your detailed Financial Disclosure statement. This is published with the article, therefore should be completed in full sentences and contain the exact wording you wish to be published.

   i). State the initials, alongside each funding source, of each author to receive each grant.

   The Funding statement has been separated out from the Acknowledgements into its own section (lines 485 – 494). All funding sources have been attributed to the specific individuals using their initials.
7. Please amend your detailed Financial Disclosure statement. This is published with the article, therefore should be completed in full sentences and contain the exact wording you wish to be published.

   i). State what role the funders took in the study. If the funders had no role in your study, please state: “The funders had no role in study design, data collection and analysis, decision to publish, or preparation of the manuscript.”

   ii). If any authors received a salary from any of your funders, please state which authors and which funders.

   The financial disclosure statement has now been updated to exactly match this statement (lines 250 – 251).

**Reviewer comments**

- Please change *P. ovale* to *P. ovale* sp. - you do not differ between *P. ovale curtisi* and *P. ovale wallikeri*..

We have changed *P. ovale* to *P. ovale* sp. throughout the manuscript. We have also included an extended description of the effects of our assays on *P. o. curtisi* and *P. o. wallikeri* in the methods (lines 181 – 183).

- Moreover the shorten forms for parasite species (like Pf) might not be in accordance to the authors guidelines - please check.

Different parasite species are referred to repeatedly throughout the text. As such, it is important to use sensible abbreviations. We use *Pf* as an abbreviation for *P. falciparum*, defined upon first usage in the text. And similarly for other *Plasmodium* species.

The appropriate sections of the author guidelines are as follows:

**Abbreviations**

Define abbreviations upon first appearance in the text.

Do not use non-standard abbreviations unless they appear at least three times in the text.

Keep abbreviations to a minimum

**Species names**

Write in italics (e.g., *Homo sapiens*). Write out in full the genus and species, both in the title of the manuscript and at the first mention of an organism in a paper. After first mention, the first letter of the genus name followed by the full species name may be used (e.g., *H. sapiens*).

**Methods**

- Reviewer #1: This manuscript written by Holzschuh et al. investigates human *Plasmodium* species coinfections, its impact on gametocyte production and parasite densities. The manuscript is very clearly written, and well structured.

In detail: Objectives are clearly stated and the methods are well described. Statistical methods are well explained and adequate.

Overall: information given in the main text is rather short, but all of the information can be found in the supplement. I recommend to add more information to the main text, so that the reader does only have to go for the supplement for very detailed information (short information on villages, on sample collection, i.e. filter paper, volume of blood used).

We thank the reviewer for their encouraging comments and noting that the manuscript is clearly written and well structured. On the specific point of moving some information from the Supplementary Information to the main manuscript, we respectfully disagree. With such a rich dataset, there is inevitably a large amount of complex information, not all of which can be incorporated in the main manuscript. We feel that we have struck an appropriate balance between providing the key information in the manuscript, and providing detailed additional information in the supplement.

- Reviewer #2: The study is clearly articulated and the study design is appropriate with also clear hypothesis. The sample size is also well sufficient.

We thank the reviewer for these supportive comments.

- Reviewer #3: Methods are sound. There are no concerns about the methods or ethical concerns; the data is thoroughly analyzed and interpreted with caution. The major achievement of the authors, developing sensitive methodologies for gametocyte carriage of all four species is an important one and makes this article of great value.

We thank the reviewer for these supportive comments.

**Results**

- Reviewer #1: The results are clearly presented and the figures are of sufficient quality.

We thank the reviewer for these supportive comments.

- Reviewer #1: One point is confusing: There are 505 pre-treatment samples mentioned, but according to methods only 504 children are included. Please explain the discrepancy.

This is a very good observation. In the study 504 children were included in the longitudinal cohort. Before treatment was administered, there was one child for which we had two samples, resulting in a total of 505 samples. This duplicated sample was removed, and we performed an updated analysis of 504 samples from 504 children. This has caused some very minor changes to some of the numeric results. None of these changes were substantial or caused any statistically significant changes.

- Reviewer #1: Many results are only presented in the supplement. If this is not a requirement I suggest to add more results to the main text, e.g. the tables stating the positivity for Plasmodium species (asexual/sexual) for the different analysis.

As noted in the general point above, we feel that we have struck an appropriate balance between the amount of information in the manuscript and in the supplement. Regarding the specific point of the positivity for Plasmodium species, this information is presented in Figure 1 of the main manuscript.

- Reviewer #2: The results are missing a clear description or statment of some data: the *Plasmodium* species blood-stage infections, and Pf and Pv gametocytes (see general comments).

  We have made efforts to provide clear descriptions of the data throughout the methods and results, most notably in Figure 1 where an intuitive overview of asexual parasite and gametocyte prevalence and density are provided.
- Reviewer #3: Figures 1-3 are clear and highly valuable. The Venn diagram nicely illustrates the richness (and complexity) of the data.

We thank the reviewer for these supportive comments.

- Reviewer #3: Figure 4 is nice but somewhat misleading since all effects, even those that are not statistically significant, are depicted. It is unclear what the grey scare for Po gametocyte density vs Pv asexual density means. I would suggest to only present colors for those associations approaching statistical significance. 0.05 is obviously not sacred and it would be acceptable to only those associations with, for example, p<0.1. Now the light green suggests, for example, a increased density but it is never statistically significant and the effect size is very small. That may lead to wrong interpretations. Alternatively, the number of observations on which the effect size is based may be presented in the cells.

  We have updated the legend of Figure 4 to state that all estimated effects are presented, regardless of statistical significance. We have highlighted that the orange squares show significant associations at a P value = 0.05, and the red squares show significant associations after correction for multiple hypothesis testing.
- Reviewer #3: Figure 5 should include number of observations.

We have included the number of observations in the legend of Figure 5.

**Conclusions**

- Reviewer #1: The conclusion section is also well written and is supported by the data. Limitations of the study are described. One additional limitation that should be mentioned is the paucity of positive samples for *P. ovale*, so that it is difficult to draw conclusions. (This might go too far – but - In addition, as it is now widely accepted in the community that *P. o wallikeri* and *curtisi* are two distinct species, that are sympatric and not recombining, and therefore one might have to consider them separately, then it is even less samples).

  This important limitation has now been mentioned in the discussion (lines 451 – 453).
- Reviewer #2: The study general conclusion is supported by the data presented but the limitations should be improved with the specification of the co-infections densities and Pv and Pf gametocytes. The age of the samples could also be limiting for an actual application for reflexion of the results.

The co-infection densities of all species of gametocytes have been summarized in Supplementary Table S12. The age of the samples has been noted as a limitation in the discussion (line 444).

- Reviewer #3: The discussion is balanced. Authors do not over-interpret their findings.

We thank the reviewer for these supportive comments.

**Summary and General Comments**

- Reviewer #1: This manuscript presents very well conducted and written work on human *Plasmodium* species coinfections, its impact on gametocyte production and parasite densities. A novel PCR for detection of Po and Pm gametocytes has been established and is presented. I recommend to publish the manuscript.

We thank the reviewer for these supportive comments.

- Reviewer #2: The paper is presenting data on “Co-infection of the four major *Plasmodium* species: effects on densities and gametocyte carriage”. As described in the methods section, 504-505 children aged 5-10 years were actively monitored for infection by qPCR and illness for 32 weeks and blood samples were collected every 2 weeks for the first 12 weeks and every 4 week 14-32. While the topic and results are very interesting, there is an important lack of information of the data used, and also discussion of the results using the sampling structure.

These are my comments:

It is not clearly specified in the paper if the *Plasmodium* species blood-stage infections, and Pf and Pv gametocytes were analysed one more time together with Po and Pm gametocytes using the same RNA extraction. As the study is using more than 10 years old samples, and the same samples being used in different publications, the authors should clearly specify the exact data newly done in the current study and those taken from old studies.

The information in methods section and supplementary data are showing that only Pm and Po gametocytes study were newly done. The authors need to clarify if they extracted again the RNA or they used left over RNA from previous studies.

We have clarified this point on lines 165 - 168 as follows:

Previously published data was incorporated in the present analysis, namely qPCR-based and qRT-PCR-based detection of *Pf*, *Pv*, *Pm* and *Po* parasitemia, as well as *Pf* and *Pv* gametocytemia. Data on *Pm* and *Po* gametocytemia was newly generated by RT-qPCR using previously extracted RNA samples stored at -80C.

In case all analysis were repeated again with newly extracted RNA, the obtained co-infections parasitemia data and Pv, Pf gametocytes data should be discussed with the old data from the same authors.

Please see above. We have not repeated RNA extraction owing to limitations in starting material

My second main comment is why the authors did not discuss the data using the sampling structure: a 32 weeks retrospective sampling was done with 9 to 10 sample collection per child. It would have been very interesting to analyse and discuss the co-infections pattern of the same sample source during the 32 weeks.

This is an excellent observation, and the authorship team have previously had substantial discussions on this point. The key analyses presented are essentially cross-sectional, and do not take full advantage of the rich longitudinal structure of the data. The reasons for this are due to the technical complexity of developing longitudinal co-infection models. We felt that the development of statistical models for cross-sectional data more than merited publication in their own right when combined with the details of the newly developed molecular assays. The challenging work of extending these statistical methods to account for longitudinal data is currently under way.

The obtained co-infections of the 4 plasmodium species in PNG are for more than 10 years again. These data should be discussed using data on plasmodium evolution in PNG.

We have added an additional statement to note with citations the high level of malaria transmission in the region at the time of the study (lines 151 – 152).

Line153: please check the sample number. 505 is used in the results

This a good observation. As also discussed in the response to reviewer #2, there was a discrepancy in our sample numbers due to two samples being included from one individual. We have now revised our analyses with 504 samples from 504 individuals.
